# Antimicrobial Resistance in Horses

**DOI:** 10.3390/ani10071161

**Published:** 2020-07-09

**Authors:** Amir Steinman, Shiri Navon-Venezia

**Affiliations:** 1 Koret School of Veterinary Medicine, The Hebrew University of Jerusalem, Rehovot 7610001, Israel; 2 Department of Molecular Biology and the Adelson School of Medicine, Ariel University, Ariel 4077625, Israel

Antimicrobial resistance (AMR) is an increasingly recognized global public health threat to the modern health-care system that could hamper the control and treatment of infectious diseases [1]. Microorganisms may serve as a reservoir for AMR in all ecological niches; therefore, a “one health” coordinated multisectorial approach is desired to investigate and address this warning phenomenon [2]. This approach appears to be a winning strategy to combat and reduce the burden of AMR, but it requires combined forces and resources that are consistently and effectively implemented by both human and veterinary health professionals [1].

Horses are among the most central animals in human history; they have been used in wars, as a means of transport, and even facilitated work in mines. Since then, the rate of contact between domesticated horses and humans has steadily increased. Nowadays, horses play an important role as sport animals and in animal-assisted therapy. Due to these close human-horse interactions, the adequate detection of infectious diseases and AMR that may affect both humans and horses is crucial, especially in cases of highly transmissible diseases [3]. Numerous important antibiotic-resistant zoonotic pathogens have been reported from horses, including extended-spectrum beta-lactamases (ESBL)-producing *Escherichia coli*, methicillin-resistant *Staphylococcus aureus* (MRSA), and multidrug-resistant (MDR) *Salmonella*. These reports have attracted increasing attention to the threat of AMR in horses [4].

During the last two decades, researchers have generated a vast amount of information on the importance of MRSA in horses, which has been recognized as an occupational risk for veterinary professionals [5]. MRSA outbreaks affecting both horses and personnel were reported from different geographic locations and reciprocal animal-personnel transmission of infections was demonstrated. Furthermore, it was previously demonstrated that on-admission MRSA colonization in horses is a risk factor to develop MRSA infection [6]. In spite of the accumulating data on the prevalence, risk factors for colonization, and resistance genes of ESBL-producing *Enterobacteriaceae*, data that links between resistant gram-negative gut colonization and equine health is still lacking. 

The occurrence of AMR pathogens causing infections in equine populations increases concern over the issue of antimicrobial stewardship that involves the judicious use of antimicrobials balanced with the requirement to treat the presenting clinical condition [7]. The challenges in equine practice include the size and value of the patient, correct and timely pathogen identification, and its susceptibility profile, together with the limited number of drugs and their indiscriminate use by clients [7]. Therefore, it is crucial to promote antimicrobial stewardship, not just among academics, public health personnel, and specialists, but also among primary care equine clinicians and equine caretakers [8].

Another important aspect of AMR in horses is the proper use of critically important antibiotics (CIA) such as fluoroquinolones, third and fourth generation cephalosporins, and macrolides. The prophylactic use of macrolide with rifampin in foals suspected to be infected with *Rhodococcus equi* has been shown to promote MDR in both *R. equi* and in gut commensals, increasing the risk of environmental shedding [9]. Disease-specific practice guidelines are required to reduce CIA use for skin, respiratory, and postsurgical infections in equine medicine [10]. Therefore, as equine practitioners and researchers, we should pay attention to the use of CIAs in equine patients treatment [1].

The aim of this special issue on AMR in horses was to collect the most recent data on the prevalence, risk factors, and characterization of different MDR pathogens in different equine cohorts from various countries. Data from Israel reports on colonization with ESBL-producing *Enterobacteriaceae* in foals on admission and in the hospital setting. ESBL colonization in neonatal foals was associated with umbilical infection and ampicillin treatment during hospitalization [11]. In Israel, risk factors for ESBL-E shedding in farm horses included horses’ breed, sex, and previous antibiotic treatment [12]. In a similar cohort of healthy horses from Canada, the number of staff members and equestrian event participation were identified as risk factors for MDR *E. coli* shedding [13]. In a study from Japan, healthy racehorses were reported to be colonized with MDR ESBL/AmpC-producing *Klebsiella pneumoniae* [14]. Another unique horse population that AMR pathogens were recovered from was equine destined for human consumption in Spain, in which both nasal and fecal carriage of a highly virulent MRSA was detected [15]. 

In addition, ESBL-producing *Enterobacteriacae* pathogens were also reported as causative agents of clinical infections in horses. In France, the percentages of MDR *Staphylococcus aureus* and MDR *Enterobacter* spp. strains causing clinical infections increased significantly during a 3-year period [16]. In Austria, MDR*Klebsiella* species were isolated from clinical samples, displaying a variety of resistance and virulence genes [17]. In a clinical bacterial collection from Texas-A&M, ESBL-producing *Enterobacteriacae* were reported with the first report of *E. coli* ST1308 in horses [18]. We believe that the new data reported here is highly relevant from a ‘’one health’’ perspective; it will help to improve our knowledge related to the issue of AMR worldwide and will assist in improving control measures, optimize appropriate therapy, and will encourage further studies in this important field. 
